# The Cost Consequences of the Gold Coast Integrated Care Programme

**DOI:** 10.5334/ijic.5542

**Published:** 2021-09-15

**Authors:** Lauren Ward, Anne McMurray, Chi Kin Law, Gabor Mihala, Martin Connor, Paul Scuffham

**Affiliations:** 1 Centre for Applied Health Economics, Menzies Health Institute Queensland, Griffith University, Brisbane, Australia; 2 School of Nursing and Midwifery, Menzies Health Institute Queensland, Griffith University, Queensland, Australia; 3 NHMRC Clinical Trials Centre, Faculty of Medicine and Health, University of Sydney, Camperdown, New South Wales, Australia; 4 Menzies Health Institute Queensland, Griffith University, Queensland, Australia

**Keywords:** integrated care, primary care, acute care, health reform, Australia, economic evaluation

## Abstract

**Introduction:**

The Australian Gold Coast Integrated Care programme trialled a model of care targeting those with chronic and complex conditions at highest risk of hospitalisation with the goal of producing the best patient outcomes at no additional cost to the healthcare system. This paper reports the economic findings of the trial.

**Methods:**

A pragmatic non-randomised controlled study assessed differences between patients enrolled in the programme (intervention group) and patients who received usual care (control group), in health service utilisation, including Medicare Benefits Schedule and Pharmaceutical Benefits Scheme claims, patient-reported outcome measures, including health-related quality of life, mortality risk, and cost.

**Results:**

A total of 1,549 intervention participants were enrolled and matched on the basis of patient level data to 3,042 controls. We found no difference in quality of life between groups, but a greater decrease in capability, social support and satisfaction with care scores and higher hospital service use for the intervention group, leading to a greater cost to the healthcare system of AUD$6,400 per person per year. In addition, the per person per year cost of being in the GCIC programme was AUD$8,700 equating to total healthcare expenditures of AUD$15,100 more for the intervention group than the control group.

**Conclusion:**

The GCIC programme did not show value for money, incurring additional costs to the health system and demonstrating no significant improvements in health-related quality of life. Because patient recruitment was gradual throughout the trial, we had only one year of complete data for analysis which may be too short a period to determine the true cost-consequences of the program.

## Introduction

The Australian Gold Coast Integrated Care (GCIC) programme began in 2014 as a four-year pilot, proof-of-concept study to examine integrated care as a solution to inequitable, fragmented, costly, and unsustainable health services; these are significant issues for Australia as well as for other countries, given population ageing and substantial growth of chronic diseases [1,2,3,4,5]. Fragmentation of services is the most significant impediment to managing chronic diseases in the Australian health care system, due to the complex interplay of funding and divided responsibilities between the federal, state/territory, and local governments for both private and public health services. Discontinuities between general practice and acute care services are also pervasive [6,7]. The need for better integration of care in Australia has been addressed in national and state initiatives aimed at linking sectors of the health care system, but to date, there have been no consistent approaches to linking primary health care with other health services [6,8]. As a time and funding-limited, proof-of-concept trial we were unable to conduct a ‘whole-system’ change. Instead the trial introduced a ‘cross-system’ approach to link primary and secondary care.

Australia has a fee-for-service model whereby primary care providers are rewarded for the quantity, rather than the quality of services. This can be a disincentive for collaborative care. Primary care efficiencies have in some cases, been responsible for reducing hospital activities, unintentionally penalising good practice [9]. From the patient’s perspective, those with complex or chronic diseases often experience a range of unmet needs as they typically have to access sequential or simultaneous services from multiple providers in different locations with varying culturally appropriate care provisions. They are also vulnerable to system barriers that obstruct their transitions through the system where there is a lack of structures or clinical governance systems that would support service integration, such as unreliable referral systems, inconsistent eligibility criteria, and minimal electronic records or secure systems for information sharing [10]. This leads to costly, poorly coordinated services with inadequate communication from care providers [11,12]. Some of these issues can be addressed through patient-centred, integrated care, where partnerships between health professionals and patients can lead to mutual, holistic understandings of patient needs, values and choices for care, balanced with caregiving approaches that meet organisational and system requirements [13,14].

The Australian National Health and Hospitals Reform Committee has strongly recommended three immediate and crucial responses to redress these issues: (1) a focus on access and equity, (2) vertical and horizontal service integration, and (3) development of an agile, self-improving, and sustainable health system focused on primary health care [15]. These systemic improvements can be met within an appropriate and adaptable model of integrated care, defined by the WHO [16] as the organization and management of health services so that people get the care they need, when they need it, in ways that are user-friendly, achieve the desired results and provide value for money. The WHO (2018) extended their expectations of integrated care with a Framework on Integrated People-centred Health Services. The framework revolves around creating an enabling environment within which services are coordinated within and across sectors, strengthening governance and accountability, reorienting the model of care, and engaging and empowering people and communities [17]. The GCIC model was built on a commitment to optimise patient-centred care by putting patients at the centre, rather than the margins of health decision-making [13]. We used Faber et al.’s (2014) three levels of involvement: *communication* at the point-of-care aimed at fostering health literacy; *consultation* to gather direct patient input such as satisfaction and quality-of-life; and *participation*, including ongoing discussion of service and information needs [14]. Integrated care is central to the National Chronic Disease Strategy and the Queensland Strategy for Chronic Disease, both of which have allocated substantial investments to meet the needs of those with chronic and complex conditions [18,19]. A 2012 national agreement between Australian federal, state, and territory governments also identified the need to address the complex interplay between different levels of government, between public and private insurers, and between acute and primary care. This agreement was an attempt to improve both health outcomes and sustainability of the Australian health system [20].

The Gold Coast in Queensland has an older population that is rapidly growing at a greater rate than the rest of the country [21,22]. It was therefore an ideal context to explore the viability of an integrated care programme targeting the growing, older Gold Coast population at highest risk of hospital admission due to chronic conditions. One of the objectives of the programme was to test the cost consequence of integrated care in a defined population with a view to making decisions on scaling the programme for a broader region. After consultation involving the community, health service planners/administrators, and local medical/health professionals the programme was collaboratively developed and funded by the Gold Coast Hospital and Health Service (GCHHS), the Gold Coast Primary Health Network (GCPHN), and Queensland Health (QH) in partnership with Griffith University (GU), where the evaluation was conducted by the Centre for Applied Health Economics.

### The GCIC programme and evaluation

The development and structure of the programme was previously reported [23]. Major constituents were general practitioners (GPs) from 15 local practices who had responded to a request for local GPs to participate as ‘network practices’ in the programme (‘network’ practices), clinicians from the GCHHS and community health services, and a multidisciplinary team of clinicians and Nurse Navigators (NNs) located in the ‘GCIC Coordination Centre’ (CC). As collaborators in the trial they were committed to help bridge primary and secondary care for those with chronic, complex, and comorbid conditions at high risk of hospitalisation. Each NN acted as a liaison between the CC and the general practice, ensuring the flow of information and services between health professionals at each setting. The goal was to produce the best patient outcomes at no additional cost to the healthcare system by supporting the GPs’ with patient management. This was intended to enhance patient experience, improve population health, reduce costs, and improve the work life of health care providers [24]. Programme features included (a) patient risk stratification, (b) individual and flexible shared care plans, collaboratively developed by the patient’s GP, the MDT members at the Coordination Centre and the patient (c) proactive general practice care, (d) holistic assessment and care provided through the CC, (e) a single point of telephone contact for patients, staff, and family members, (f) rapid access to home services and specialist teams, (g) enhanced information and communications technology through an electronic shared care record database, and (h) shared decision-making between patients, carers, family members, general practice staff and clinicians of the CC [23]. Care-as-usual for chronic disease patients (those in the control group) consisted of accessing primary care through their usual GP. and for an emergency patients would access hospital services directly through the emergency department of the hospital or by referral from the GP to specialists or outpatient services. Programme planning began in 2014, followed by a three-year participant recruitment (2015–2017). All components of the programme were fully implemented between October 2017 and September 2018, which represents the intervention period used for analysis and reported here. Throughout this period all collaborators, including the GPs, met on a regular basis to discuss the programme and its progress.

The economic evaluation used a cost-consequence analysis framework to assess whether the programme outputs could be achieved without incurring additional costs. The evaluation required a pragmatic non-randomised controlled clinical trial to examine the following outcomes: (a) hospital utilisation: GCHHS episodes (inpatient admissions, emergency department attendances, and outpatient attendances); and costs (expenditure), (b) Medicare Benefits Schedule (MBS) and Pharmaceutical Benefits Scheme (PBS) claims and expenditure (benefits paid), (c) patient reported health outcome measures (PROMs) including quality of life, and (d) patient and staff satisfaction with care. The evaluation protocol has been previously published [25]. Ethics approval was received from the GCHHS (HREC/15/QGC/22) and GU (MED/22/15/HREC), as well as QH Public Health Act approval (RD005624), and the Commonwealth Department of Human Services.

## Methods

### Target population

Patients of the GCHHS and those affiliated with participating practices determined to be high risk, that is, those with complex and co-morbid conditions considered high users of hospital services, were identified for inclusion in the programme through risk stratification, including GP and clinician judgement (Supporting Text 1).

### Study perspective

This paper reports the programme evaluation from a healthcare system perspective. The GP, patient and staff perspectives on the programme are reported in a previous publication [26]. Data on private hospital events and ‘out-of-pocket’ costs were not collected, as access to private hospital data was not available at the time of evaluation and administering a separate survey to collect ‘out-of-pocket’ costs was deemed too much of a burden for the patients.

### Comparator

A group of participants were identified from GCHHS utilisation data to act as a control group who received usual care. This involved a two-step process: initial identification and case-control matching (Supporting Text 2). The aim was to achieve the best possible match on the basis of patient level hospital data. A sub-group of control group patients were randomly selected from the initially identified patient group and invited by mail to participate as an ‘active’ control to provide parallel PROM data to the programme participants. The remaining control participants were seen as the ‘passive’ control group as their information was collected from hospital administrative data only.

### Data collection and outcomes

The analysis of healthcare utilisation included episodes per patient-year and expenditure per patient-year for the pre-enrolment period (July 2012 to February 2015) and for the full programme implementation period (October 2017 to September 2018). Data were collected from the following sources: Healthcare utilisation included: GCHHS events (emergency department attendances; hospital inpatient episodes, including potentially preventable hospitalisations (PPHs) [27] and length of stay; and outpatient appointments); GCHHS utilisation costs; and MBS/PBS claims and benefits paid.

PROMs were collected at baseline, 12-month, 24-month, and 36-month follow-up through participant surveys capturing the quality of life (AQoL-4D [28]), social support (LSNS-6 [29]), capabilities (ICECAP-O [30]), satisfaction with care (SAPS-7 [31]), and chronic illness care (PACIC [32]) aspects of their healthcare experience. These instruments were selected to cover the spectrum of quality of life across a wide range of health states, social support networks, functional capability, and satisfaction with care in a population. Participants’ AQoL-4D scores were converted to health utility index, where 1 = perfect health and 0-dead, according to the algorithm derived by the Centre for Health Economics of Monash University [33], and ICECAP-O scores converted to a 0–1 scale using the UK algorithm [30]. MBS/PBS and survey data were only collected for the active controls [25]. Participants were studied for 12–36 months from enrolment depending on date of consent, unless they left the study early due to withdrawal, moved away from the Gold Coast area, moved to a retirement home, or death. Although the duration of the study was four years, the evaluation only included a full 12 months of data due to the time taken to recruit patients into the programme. Healthcare utilisation excluded cancer-related attendances, such as outpatient appointments for oncology, emergency presentations due to cancer, inpatient admissions due to cancer diagnoses (ICD-10 codes C00-C42, C45-C97, and D37–48), MBS claims for radiotherapy, and PBS claims for anti-neoplastic drugs. Inpatient admissions due to skin cancer were, however, included.

### Data analysis

The programme effect on healthcare utilisation was assessed with the difference-in-difference technique, and with a generalised linear model with log link [31]. To examine the difference in healthcare utilisation between intervention and control groups, the monthly number of GCHHS events and MBS/PBS claims was taken as the dependent variable, and a Poisson regression model was chosen in the generalised linear model. To examine the programme effect on participants’ monthly healthcare expenditure and inpatient length of stay, a log-normal model was chosen in the generalised linear model. A value of 0.01 for events or costs was added to programme participants who had zero healthcare utilisation during the period as the log of zero is undefined.

A retrospective analysis of total healthcare expenditure per-patient per year (i.e. GCHHS and MBS/PBS costs) for each study group was calculated and compared between the pre-enrolment period and the full programme implementation period, by dividing total healthcare expenditure over each period by the corresponding number of participants. All expenditures were standardised to September 2018 Australian Dollar values based on the health price indices from the Australian Institute of Health and Welfare [34]. Due to the short programme implementation period (Oct 2017-Sep 2018; 12 months), a discount rate was not applied in the analysis.

We excluded inpatient episodes with costs or length of stay deemed as outliers (outliers were identified using the three standard deviations method) [35]: inpatient episodes with adjusted inpatient expenditure of more than AUD$56,935 or with a length of stay of more than 35.36 days. These accounted for about 0.15% of the data sample.

PROMs were compared with population norms, with the difference between intervention and control groups over time assessed using the difference-in-difference approach. Length of exposure to the programme was adjusted as one of the covariates in the model. The intervention effect on mortality risk was assessed by Cox regression. Survival time of participants (in days) was defined as the time difference between their enrolment in the GCIC evaluation and event (death or censoring) date.

All analyses were performed using SAS version 9.4 M5 [36], and p-values of less than 0.05 were considered statistically significant. Missing values were not imputed.

## Results

### Recruitment

**Figure 1:**
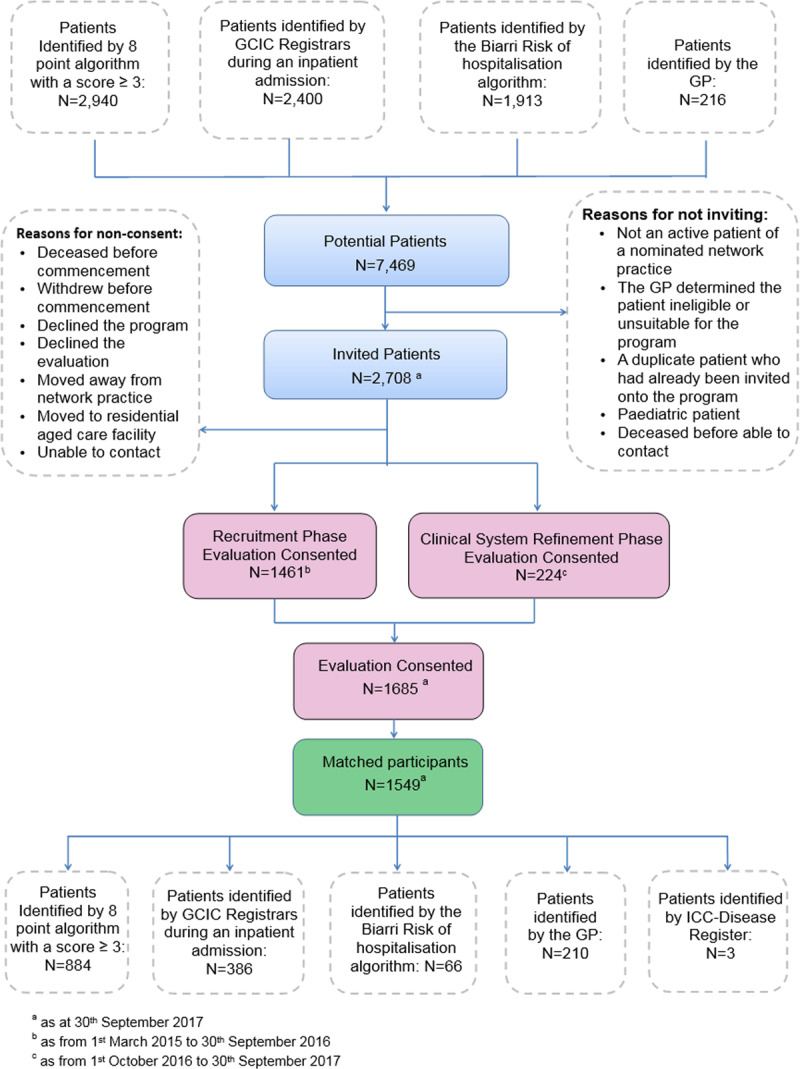
Recruitment flowchart (intervention group).

Of the 2,708 patients invited into the programme, 1,795 (66%) consented to the programme and 62% (n = 1,685) consented to participate in the evaluation (***Figure 1***). A total of 1,549 programme participants were successfully matched to 3,042 control participants and included in the final analysis. From those identified as potential controls, 4,032 were invited to participate in the evaluation as active controls, with 22% (n = 868) consenting and completing the baseline questionnaire and agreeing to continue providing data for the evaluation.

***Table 1*** outlines participant baseline demographics. Intervention and control participants were generally comparable, however there were some baseline demographic differences which were adjusted in the analysis.

**Table 1:** Participant baseline characteristics; intervention and control groups.


	INTERVENTION n = 1,549 (%)	ACTIVE CONTROL n = 868 (%)	p-VALUE/PASSIVE CONTROL (n = 2,174)

Age category (in 2015):			p = 0.259*

up to 54 years	225 (15)	136 (16)	294 (14)

55 to 74 years	684 (44)	430 (50)	990 (46)

75 years and above	640 (41)	302 (35)	890 (41)

Gender:			p = 0.895*

female	822 (53)	414 (48)	1,194 (55)

male	727 (47)	454 (52)	980 (45)

Aboriginal and/or Torres Strait Islander (*N* = 2,181)	16 (1)	16 (2)	p = 0.043

Employment status (*N* = 2,205):			p < 0.001

retired	1,043 (72)	506 (66)	

permanently ill/unable to work	153 (11)	89 (12)	

employed (full/part-time/casual)	126 (9)	110 (14)	

unemployed	90 (6)	21 (3)	

home duties/carer	22 (2)	28 (4)	

student	9 (1)	8 (1)	

Participant lives with (*N* = 2,090):			p = 0.451

spouse	726 (53)	392 (55)	

alone	373 (27)	191 (27)	

other family members	279 (20)	129 (18)	

Has a carer (*N* = 2,209)	454 (31)	207 (28)	p = 0.118

Carer lives with the participant (*N* = 662)	374 (81)	167 (84)	p = 0.338

Participant is also a carer (*N* = 2,190)	123 (8)	68 (9)	p = 0.451

Smoking status (*N* = 2,226):			p < 0.001

current smoker	210 (14)	73 (10)	

stopped	569 (38)	399 (54)	

never smoked	702 (47)	273 (37)	

Highest level of education (*N* = 2,147):			p < 0.001

up to Grade 12	1,095 (78)	444 (60)	

TAFE certificate, trade, or apprenticeship	212 (15)	222 (30)	

university/postgraduate	99 (7)	75 (10)	

Total annual household income before tax (*N* = 1,918):			p < 0.001

$39,999 or less	1,094 (84)	456 (74)	

$40,000 to $59,999	164 (13)	87 (14)	

$60,000 to $79,999	25 (2)	29 (5)	

$80,000 or more	22 (2)	41 (7)	

Private health insurance (*N* = 2,197)	293 (20)	166 (22)	p = 0.303

Private health insurance with hospital cover (*N* = 442)	239 (85)	140 (88)	p = 0.427

Private health insurance with extras cover (*N* = 425)	226 (82)	112 (75)	p = 0.067


Total GCHHS episodes for the intervention and control groups for the period July 2012 to September 2018 included 29,611 emergency presentations, 149,812 outpatient appointments, and 67,070 inpatient admissions. From a total of 61,011 GCIC inpatient admissions, 11% (n = 6,520) were categorised as potentially preventable hospitalisation (PPH).

The incremental change in average number of episodes per person per year for all GCHHS services (inpatient, outpatient, and emergency), between the pre-enrolment period and full programme implementation period was higher in the intervention group compared to the control group. For example, the difference in emergency attendances and inpatient episodes between groups increased by approximately 50% over time, while the increase in outpatient attendances almost doubled for the intervention group compared to the control group. Greater increases in length of stay (LoS) between pre-enrolment and full programme implementation were also evident for the intervention group compared to the control group (an average of 4.19 more days for all inpatient episodes and 0.87 more days for PPHs for the intervention group).

While the increase in the average number of MBS claims per person per year between the pre-enrolment and full programme implementation periods was similar for both groups, the increase in the average number of PBS claims for the control group was close to double that of the intervention group over the same period (13.9 more PBS claims per year were made by control patients than intervention patients at full programme implementation).

GCHHS expenditure for both groups increased between pre-enrolment and full programme implementation periods, however, the increase was greater for the intervention group. For example, inpatient services for the intervention group increased by AUD$6,700 more than the control group between periods.

### MBS/PBS Claims and Expenditure

In contrast, the incremental change in per person per year MBS/PBS claims expenditure was AUD$1,500 greater for the control group compared to the intervention group between periods; of which $1,480 was for PBS claims expenditure.

Total average per person per year healthcare expenditure during the full implementation period for the intervention group was AUD$28,720; $6,400 greater than the control group during the same period. The cost of providing the GCIC programme during the same period was $8,700 per person per year. Thus, the total per person per year cost in the intervention group was $15,100 higher than the control group (***Table 2***).

**Table 2:** Per person per year episodes/claims, inflation-adjusted per patient per year healthcare expenditure (AUD$) and per patient GCIC programme cost by study group.


PER PERSON PER YEAR	TYPE OF HEALTHCARE	PRE–ENROLMENT				FULL PROGRAMME IMPLEMENTATION				
	
		INTERVENTION		CONTROL		INTERVENTION			CONTROL	
			
		AVERAGE	%	AVERAGE	%	AVERAGE	%^a^	%^b^	AVERAGE	%

**Healthcare Utilisation**										

GCHHS Events	Emergency department attendances	0.9	–	0.8	–	1.8	–	–	1.2	–

	Outpatient visits	4.1	–	3.4	–	8.4	–	–	5.8	–

	Inpatient admissions	1.5	–	1.6	–	3.4	–	–	2.8	–

	PPH^c^	0.2	–	0.2	–	0.4	–	–	0.3	–

LOS (days)	Inpatients	3.88	–	4.53	–	8.75	–	–	5.21	–

	PPH^c^	0.46	–	0.6	–	1.64	–	–	0.91	–

MBS/PBS Claims^d^ (n)	MBS	53.2	–	50.4	–	82.3	–	–	77.6	–

	PBS	57.7	–	53.6	–	79.7	–	–	93.6	–

**Healthcare Expenditure (AUD$, Sept 2018)**										

GCHHS	Emergency	740	5.0	690	4.6	1,680	5.8	4.5	1,120	5.0

	Outpatients	1,830	12.3	1,460	9.8	3,170	11.0	8.5	2,150	9.6

	Inpatients	7,300	49.0	8,040	54.1	16,430	57.2	43.9	10,470	46.9

	PPH^e^	910	–	1,120	–	3,130	–	–	1,710	–

	**Sub–total GCHHS expenditure**	9,870	66.2	10,190	68.6	21,280	74.1	56.9	13,740	61.6

MBS/PBS Benefits paid	MBS	2,920	19.6	2,750	18.5	4,110	14.3	11.0	3,960	17.7

	PBS	2,110	14.2	1,920	12.9	3,330	11.6	8.9	4,620	20.7

	Sub–total MBS/PBS benefits paid	5,030	33.8	4,670	31.4	7,440	25.9	19.9	8,580	38.4

**All healthcare expenditure (AUD$)**		14,900	100.0	14,860	100.0	28,720	100.0	76.8	22,320	100.0

GCIC expenditure (AUD$)		–	–	–	–	8700	–	23.2	–	–

**Total healthcare expenditure (AUD$)**		14,900	100.0	14,860	100.0	37,420	100.0	100.0	22,320	100.0


PPH = potentially preventable hospitalisations; GCHHS = Gold Coast Hospital and Health Service; LOS = Length of stay; MBS = Medicare Benefits Schedule; PBS = Pharmaceutical Benefits Scheme; ^a^ Proportion of healthcare costs excluding GCIC program cost for the intervention group; ^b^ Proportion of healthcare costs including GCIC program cost for the intervention group; ^c^ Of all inpatient admissions; ^d^ Active control participants only; ^e^PPH expenditure is excluded from sub-total GCHHS expenditure because it is a part of inpatients expenditure; all costs rounded to nearest ten dollars and standardised to the value on September 2018.

### Programme Expenditure

Between July 2014 and December 2018, the programme expenditure was AUD$26.3 million. Labour costs accounted for approximately 85% ($22.3 million), with the leading labour cost item being managerial, clerical, and non-clinical navigation at $5.4 million (24%), followed by nursing at $4.8 million (22%) and medical at $4.3 million (19%) (***Table 3***).

**Table 3:** GCIC direct programme expenditure.


	COST ITEM	AMOUNT (AUD$)

**Labour expenses**		

	Managerial and Administration	5,353,057

	Nursing	4,820,261

	Medical	4,289,264

	Allied Health Practitioners	3,146,068

	Information and Technology, Project and Professional Staff	3,416,986

	Brokered Services	935,801

	Other Employee Related Expenses^a^	355,475

		

**Total Labour**		**22,316,912**

**Non-labour expenses** ^b^		3,947,704

**Total Expenses**		**26,264,617**


Allied Health Practitioners = occupational therapists, pharmacist, physiotherapist, psychologist, social workers; Nursing = nurse practitioner, nurse manager, nurse navigators, clinical nurse, registered nurses, enrolled nurse; Managerial and Administration = managing director, operations manager, divisional finance manager, legal officer, administration officers, service navigators; Information Technology, Project and Professional Staff = principal analysts, principal project officers, project manager, project assistant, research manager, research assistant, contractors (staff training and project development); Medical = medical directors (general physician and general practice), geriatrician, staff specialists, medical registrars; Brokered Services = includes costs associated with engagement of Allied Health Services Australia, Royal District Nursing Service (RDNS) Elan Medical Supplies, and interpreter services; Training and establishment costs written into the Standing Offer of Arrangement with RDNS; ^a^ Includes WorkCover premiums, labour related taxes, benefits, training and development; ^b^ Includes supplies, services, rent, utilities (electricity), building establishment costs.

### Results of Difference-in-Difference Analysis

#### Hospital Utilisation

Results of the difference-in-difference analysis (***Table 4***) show significantly greater increases in all GCHHS events for the intervention group compared to controls between pre-enrolment and full programme implementation periods. When separately analysed by type of GCHHS event, increases in PPHs and emergency attendances for the intervention group were 39.2% (p < 0.01) and 36.2% (p < 0.01) greater than the control group between pre-enrolment and full programme implementation periods (p < .001).

**Table 4:** Results of difference-in-difference analysis (study group x period of programme) on per patient per year episode, length of stay, expenditure, MBS and PBS use.


PER PERSON PER YEAR	TYPE OF HEALTHCARE	DIFFERENCE-IN-DIFFERENCE	p-VALUE

**GCHHS Episodes**	Emergency department attendances	0.362^a^	<.001*

	Outpatient visits	0.181^a^	<.001*

	Inpatient admissions	0.299^a^	<.001*

	PPH	0.392^a^	<.001*

**LOS (days)**	Inpatient admissions	0.673^b^	<.001*

	PPH	0.840^b^	0.023*

**Number of Claims**	MBS	0.005^a^	0.548

	PBS	–0.235^a^	<.001*

**Expenditure (AUD$, Sept 2018)**	Emergency department	0.328^b^	0.862

	Outpatient costs	0.160^b^	0.801

	Inpatient admission costs	0.547^b^	0.539

	PPH costs	0.814^b^	0.837

	MBS	–0.024^b^	0.981

	PBS	–0.420^b^	0.916


n.b. A value of 0.01 was added to GCIC Participants with zero healthcare utilization/cost; Generalised linear model (GLM) with log link function applied; *Significance p < 0.05; ^a^ Poisson regression; ^b^ Log-normal regression; PPH = potentially preventable hospitalisations; LoS = length of stay; MBS = Medicare Benefits Schedule; PBS=Pharmaceutical Benefits Scheme.

While there was no significant difference found between groups for MBS claims between the two periods, intervention participants made 23.5% fewer PBS claims than controls between pre-enrolment and full programme implementation periods (p < .001).

There were no significant differences between intervention and control groups in GCHHS expenditure or MBS/PBS benefits expenditure between the two periods.

#### Patient-reported Outcomes

No significant difference was found in AQoL-4D scores between the study groups. Intervention participants had a significantly greater reduction in scores by 0.037 points for ICECAP-O (p = 0.005), by 1.769 points for LSNS-6 (p < .001), and by 2.725 points for SAPS-7 (p < .001). In contrast, the PACIC scores increased more for the intervention group by 0.326 points (p < .001) between pre-enrolment and full implementation periods (***Table 5***).

**Table 5:** Programme effect on patient reported outcome measures, intervention, and active controls.


PATIENT-REPORTED OUTCOME MEASURE	SURVEY TIME-POINT	STUDY GROUP		DIFFERENCE-IN-DIFFERENCE	p-VALUE

		INTERVENTION	CONTROL		

AQoL-4D	Baseline	0.535 (0.300)	0.515 (0.286)	–0.03	0.117

	Last follow-up	0.502 (0.293)	0.485 (0.285)		

ICECAP-O^a^	Baseline	0.866 (0.161)	0.821 (0.155)	–0.037	0.005*

	Last follow-up	0.822 (0.171)	0.797 (0.175)		

LSNS-6	Baseline	20.4 (6.74)	18.3 (6.36)	–1.769	<0.001*

	Last follow-up	18.8 (6.32)	18.2 (6.20)		

SAPS-7	Baseline	24.5 (3.80)	21.2 (4.58)	–2.725	<0.001*

	Last follow-up	21.9 (4.10)	20.8 (4.35)		

PACIC	Baseline	2.88 (0.85)	3.08 (1.08)	0.326	<0.001*

	Last follow-up	3.23 (1.08)	3.10 (1.09)		


^a^ Participants aged 65^+^ In 2015; * Significance p < 0.05.

### Survival

**Figure 2:**
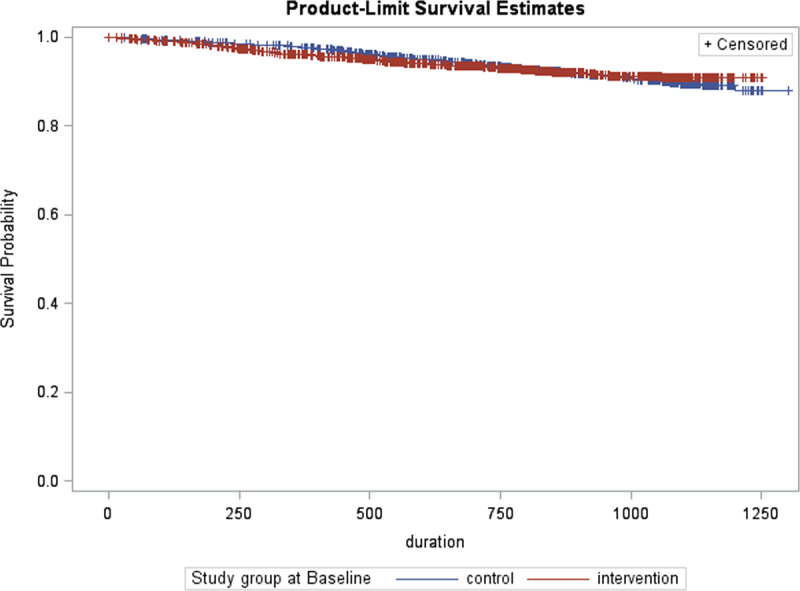
Kaplan-Meier survival function (non cancer-related deaths excluded).

One hundred and twelve intervention (7%), 66 active control (8%), and 181 (8%) passive control group participants experienced non cancer-related death during the programme. No obvious difference in the probability of survival was observed between the intervention and control groups (***Figure 2***). Results of the Cox regression model confirmed that the hazard ratio was not statistically significant (HR = 1.022, 95% CI = 0.817–1.279, p = 0.847), after adjusting for all significant covariates.

## Discussion

### Key findings

The economic evaluation clearly showed that the programme did incur additional costs to the health system. Our expectation of being able to demonstrate value for money in implementing a comprehensive integrated care programme was dispelled by the increase in hospital services used by patients enrolled in the programme. The GCIC programme was also costly to develop and implement, requiring a large number of clinical, managerial, and technical staff to oversee the development and refinement of the programme and its data linkage systems. No difference was found in health-related quality of life between intervention and control participants, indicating that both groups were receiving adequate care. Our broader analysis of patient and health provider satisfaction and perceptions of their care also showed positive results, which we report in a separate paper.

The economic findings were unexpected based on the findings of previous research [37], but evaluations of integrated care (IC) programmes come with challenges that have been acknowledged in other contexts. Most integrated care programmes are highly variable in terms of programme components and delivery, making cost comparisons elusive [1,37]. Kadu et al.’s [38] systematic review of the quality of economic evaluations in IC found that few programmes reported cost reductions. Reasons include: the breadth of services required; start-up costs such as information technology, personnel, and other resources; the need to manage multiple patients irrespective of the target group; continuous changes in care delivery; uncertainty in poorly specified outcomes; and because programmes are designed to correct underuse and ensure timely access to care, which can distort figures on service use [39,40].

Funding uncertainty is another problem previously identified in Australian studies [9]. Like the GCIC programme the Central Coast Integrated Care Programme (CCICP) in New South Wales received funding for a designated period to demonstrate how integrated care could be implemented while achieving large-scale transformation, which was part of their state health plan. The GCIC programme was introduced in a designated region of Queensland with the full commitment of those providing funding and considerable enthusiasm by local GPs, the Health Service and the PHN. Their ongoing involvement and the willingness of patients to participate in the trial were an indication of the cultural alignment required for clinical, professional and organisational integration [41]. The programme was not part of a wider system transformation but it was built on the shared assumption, common purpose and expected outcome of providing high quality, integrated care. These factors are essential in implementing highly complex, innovative interventions [42]. Integrated care researchers have suggested that in future, evaluation methodologies should aim to capture this type of cultural commitment in participatory, person-centred studies [43,44]. However, as in other programmes, it is currently the economic viability that is typically used to justify further investment by health planners [45]. Tsiachristas (2016) suggests that financial incentives may undermine intrinsic motivations, but this was not the case in the GCIC programme as the programme did not incentivise health professionals. Instead, the issue was insufficient implementation time, due to a longer than anticipated patient recruitment period, delaying implementation of the full suite of programme components. Other researchers have also found that the cost effectiveness of programmes is typically linked to having sufficient follow-up time to show the impact of IC models [4,37,38,46]. Bardsley et al. [47] evaluated eight UK programmes and found few cost savings, suggesting a need for longer follow-up to show economic responsiveness. Another broad (WHO) review of 19 IC programmes showed only one that reduced costs related to ED presentations [3]. Zulman et al. [48] explain the need for a longer duration on the basis that the trajectory of patient needs changes from high intensity in the initial stage while clinicians are building trust and helping them modify health behaviours, and any subsequent reduction in future health services utilisation.

### Healthcare service use

The substantial level of healthcare services use for GCIC programme participants also included a greater increase in the number of Potentially Preventable Hospitalisations (PPH), compared to the control group. Likewise, increases in hospital service utilisation, including PPHs, were also found in the New South Wales Health Chronic Disease Management Program (CDMP) evaluation, which they suggest could be an indication that the programme identified complex conditions or unaddressed needs that require hospitalisation [7]. We also hypothesise that the increased hospital utilisation of the GCIC participants could be because there was greater attention to patients’ self-identified needs through their 24-hour access to a nurse-monitored telephone service and home visits where necessary. As we argue in our previous report, GPs rated communication, care coordination and timeliness of services highly, despite some minor declines in the ratings over time [26].

A high rate of hospital services utilisation also underlines the need for greater proactive, preventive, and participatory community services, especially those based on teams or networks, as recommended in a recent OECD report on strengthening primary health care [49]. This issue is currently being debated in the literature in relation to identifying ambulatory care sensitive conditions that should be managed in primary care [50,51]. Primary care researchers have long maintained that patterns of accessing services are indications of a wide range of factors influenced by both population health and health system performance. Included are population factors such as socioeconomic status (deprivation); rurality and other demographics; and private insurance [52]; hospital policies such as the distribution and variability of GP practice and decisions about admissions; multiple impacts of comorbidities [50]; health system factors such as emergency department admission criteria and LoS issues; and commitment to patient-centredness as a proxy for quality care [51]. Hospital admission data is widely variable. A systematic review of patient outcomes from IC programmes in several countries showed considerable ambiguity in hospital admission data among older people, whether they were admitted in the Danish, UK, Canadian or Kaiser-Permanente system in the USA. Some programmes showed a positive impact on admissions and LoS with a single point-of-entry where case management was used, while others found the opposite effect [51]. These researchers concluded that reductions in admissions are more likely to be greater in analysing condition-specific admissions rather than all-cause admissions, as we have done [53].

Another unexpected finding was that in anticipating a reduction in hospital episodes of care, the proportion of MBS/PBS claims/costs would increase, but we found the opposite effect (see ***Table 2***). In our study, providing services at the Coordination Centre, including medication reviews, may explain the proportionately smaller increase in MBS and PBS claims for intervention patients. Although the economic value of medication reviews is yet to be demonstrated, current studies indicate that the added value of using community pharmacy services is faster access and convenience for patients leaving GPs time to focus on more complex patients [54,55]. Clearly, primary care is a more appropriate option for many patients. Kringos et al’s (2013) study of primary care systems in 31 European countries found that a widely developed primary care system has effects on the whole system and is associated with better population health, lower rates of unnecessary hospitalisation and relatively lower socioeconomic inequality. However, they also found that strong primary care can be a cost driver, as total health expenditures were higher after adjusting for national income in those countries with stronger primary care structures [56].

### Patient-reported outcomes

While there were no significant differences in survival and quality of life observed between groups, the active control group did statistically significantly better than the intervention group in self-reported capability, and social support measures. Given the age of participants in both groups, a decline in self-reported health and quality of life would be expected over time [57], however the relatively greater decrease in capability scores over time for the intervention group may be due to potential demographic differences such as income and employment [58]. While subgroup analyses might go some way to confirm possible explanations for this decrease, the risk is that any statistical inference (positive or negative) would be misleading due to the high likelihood of small numbers in each subgroup. Without longer term follow-up we tried to avoid any false expectations and conclusions. Compared to controls, the intervention group experienced a significantly greater reduction in satisfaction with care scores. However, the greater increase in patient assessment of chronic illness care (PACIC) scores for intervention participants compared to controls, indicated a higher level of satisfaction with the quality of integrated healthcare received. This finding indicates that chronic care needs were well met from the perspective of both patients and care providers [26].

### Limitations

The GCIC evaluation model adopted a pragmatic design rather than a randomised clinical trial, which has also been the case in other evaluations of chronic disease programmes [4,38,39]. The target group selection may also have been a limitation. The study sought to recruit 1,500 patients and 3,000 controls from the GCHHS patient population to recruit the 3% of patients with the most complex conditions, according to the Kaiser Permanente pyramid [59]; that is, the 3% of patients considered at the highest risk of presenting to hospital. Some researchers suggest that reducing long-term costs for high-need patients requires efforts across all risk tiers, with a focus on high and middle level risks [60]. This would require a broader range of clinical interventions to anticipate the full range of needs, and until tested in another trial, may also not be viable or cost-effective. In the GCIC study, some discretion was used, with clinician judgement identifying patients presumed to benefit from the programme. This created a more inclusive approach but created variability in the recruitment strategy by introducing a selection bias that may have represented a threat to implementation fidelity. We also found that slow progress in patient recruitment diverted resources to finding appropriate participants. Given that the trial was planned for a designated period of time, this resulted in a short 12-month period of full implementation.

Another limitation of the study included the inability to find a perfect control match for all intervention patients due to the limited pool of patients in the GCHHS system, resulting in differences in case matching. Potential intervention participants were identified from hospital data and GP clinical judgement, with control participants being case matched to intervention participants where possible. Some baseline differences were found in demographic, socioeconomic, diagnostic, and service utilisation between intervention and control groups. There was a higher number of intervention patients who lived alone, with several lower socioeconomic status indicators, such as being retired/unemployed, low education and income, and a higher rate of smoking. It would be useful to explore whether differences between groups in study outcomes could have been influenced by these variations in baseline characteristics.

### Implications for policy

As a rapidly growing region, especially for older persons, increased demand and use of health services is expected [61]. However, health service use is influenced not only by the design and availability of health care facilities, programmes, and the supply of health care providers, but by an individual’s social circumstances, determinants, and preferences. Most of these variables are highly unpredictable, which adds uncertainty for projecting patterns for the future, including those in other metropolitan areas of Australia. A cross-case analysis of international integrated care programmes has found that fully integrating data across organisational and professional boundaries with general practitioners is also challenging [4]. We were able to do this with specific project funding, but widespread health system changes may be more challenging. We were able to meet some of the goals of the Health Reform Commission [16], in improving access and equity for the intervention group and in providing horizontal integration of primary and secondary services. However, a time and funding limited proof-of-concept trial is only one step in developing an agile, self-improving and sustainable health system focused on primary care. Greenhalgh and Papoutsi’s [62] rapid review of spreading and scaling up innovation and improvement identified common reasons for the challenges, including the additional expenditures, having to divert staff from their usual work, deeply held cultural or professional norms and willingness to take risks. Supportive policies, professional buy-in, collaborative learning and strong leadership are some of the factors that can overcome barriers to scaling up interventions across the system. These elements are part of our lessons learned from the programme. One of the most important of these lessons was the need to reconsider development of the Shared Care Record. Recommendations for future programmes include the need to modify existing electronic patient records rather than develop these for a specific programme [26].

## Conclusion

The GCIC programme, in the form that was implemented, could not show value for money due to high programme development and running costs, a greater increase in hospitalisations among intervention participants, and no significant improvement in their health-related quality of life. The time to identify differences in outcomes and costs was one year, indicating that a longer follow-up period might show other outcomes.

## Additional Files

The additional files for this article can be found as follows:

10.5334/ijic.5542.s1 SUPPORTING TEXT 1. Participant identification and risk-stratification strategies.

10.5334/ijic.5542.s2 SUPPORTING TEXT 2. Control group matching process.
