# Supplementary material for: The Cost Consequences of the Gold Coast Integrated Care Programme
Source: Int J Integr Care. 2021 Sep 15;21(3):9. doi: 10.5334/ijic.5542 (PMC8447978; doi:10.5334/ijic.5542)
Supplement: SUPPORTING TEXT 1. — Participant identification and risk-stratification strategies. [file ijic-21-3-5542-s1.pdf]

The cost consequences of the Gold Coast Integrated Care Programme

SUPPORTING TEXT 1. Participant identification and risk-stratification strategies

In the first instance, risk stratification strategies to determine inclusion in the programme was based on whether or not three or more of the following eight criteria were present for an individual patient:

1. ≥ 1 inpatient admissions in the past 3 years;
2. ≥ 1 emergency department presentations in the past 3 years;
3. ≥ 5 medications;
4. ≥ 20 general practice visits (ever);
5. Coded diagnosis of diabetes, extracted from primary care clinical software;
6. Coded diagnosis of chronic heart disease, extracted from primary care clinical software;
7. Coded diagnosis of chronic obstructive pulmonary disease, extracted from primary care clinical software;
8. Coded diagnosis of chronic kidney disease, extracted from primary care clinical software.

In addition, GPs identified other patients attending their practices who were likely to benefit from the programme.
As a result of slow recruitment, additional patient identification strategies were employed in 2016:

- Patients who presented to a Gold Coast Hospital and Health Service (GCHHS) emergency department were reviewed by GCIC registrars for consideration of inclusion in the programme. Using the eight-point criteria as a general guide, patients considered suitable were informed about the programme at the bedside, and where applicable, provided consent to discuss their recruitment to the programme with their GP. The GPs were then asked to consider if they felt this patient was amenable for the programme and to notify GCIC if considered unsuitable (e.g., patient no longer attends the practice or patient not clinically suitable for the programme);
- A risk stratification algorithm, developed by commercial mathematicians, was used to predict the risk of hospitalisation (RoH) within the next 12 months for GCHHS patients affiliated with participating practices. The algorithm determined that a RoH score of >64% could serve as a baseline for considering patients amenable to GCIC enrolment. Patients with a RoH score of 70% and higher were identified and their details sent to the general practice to be considered for enrolment in the programme. In addition, the RoH score has been used as an adjunct to the hospital-based registrar reviews; Identification by the GP has been an ongoing source of new patients for the programme. With support from the GCHHS, Nurse Navigators (NN) were appointed and allocated to each participating general practice to liaise between practice staff and GCIC. GPs recommended patients to their NN on the basis of historical GCHHS utilisation data from the GCIC electronic database to guide the decision to accept the patient into programme. Consent was obtained from all GPs to invite their patients into the programme;
- Patients already enrolled in the programme could also refer family members to the programme in consultation with their GP to confirm eligibility.
