# Supplementary material for: The Cost Consequences of the Gold Coast Integrated Care Programme
Source: Int J Integr Care. 2021 Sep 15;21(3):9. doi: 10.5334/ijic.5542 (PMC8447978; doi:10.5334/ijic.5542)
Supplement: SUPPORTING TEXT 2. — Control group matching process. [file ijic-21-3-5542-s2.pdf]

The cost consequences of the Gold Coast Integrated Care Programme

SUPPORTING TEXT 2. Control group matching process

The following outlines the matching strategy used to identify control participants and is represented as a flow chart in Figure 1.

Step 1.
Intervention group: Raw data was received from GCHHS Health Analytics for 1,682 intervention participants. Those with no inpatient admissions (or missing data) during this period were ineligible for matching, therefore 133 participants were excluded. The remaining 1,549 were split into two groups: (a) 993 participants with at least one admission with primary or associative diagnoses of heart disease, diabetes mellitus (DM), renal failure or chronic lower respiratory disease and (b) the remaining 556 participants with at least one admission with other diseases. Matching was undertaken using data at baseline (recruitment) of the intervention group and then matched to hospital records for the control group.

Passive control group: De-identified raw data was received for 86,665 passive control candidates. One hundred percent of this group had at least one inpatient admission during this period and were therefore eligible for matching. The group was split into two: (a) 18,207 patients with at least one admission with primary or associative diagnoses of heart disease, DM, renal failure, or chronic lower respiratory disease, and (b) 68,458 who were admitted at least once with a diagnosis other than the above listed four primary or associative diagnoses. Matching of a control to one intervention participant was performed separately by these sub-groups: 1,205 passive controls were matched in the (a) sub-group, and 1,025 were matched in the (b) sub-group.

Active control group: The active control group was randomly selected from the control group to provide external validation to the HHS data and other measures such as quality of life. A sample of 20% was selected from the passive controls to recruit at least 600 participants for the active control group. Allowing for some deaths and losses to follow-up, over-sampling by 25% was undertaken (750). Raw data was received for 893 active control participants. Those with no inpatient admissions during this period were ineligible for matching, therefore 25 participants were excluded. The remaining 868 were split into two groups: (a) 781 participants with at least one admission with primary or associative diagnoses of heart disease, DM, renal failure, or chronic lower respiratory disease, and (b) the remaining 87 participants with at least one admission with other than the above listed diagnoses. Matching of a control to one intervention participant was performed separately by these subgroups. One hundred percent of the 868 active controls were matched to an intervention participant.

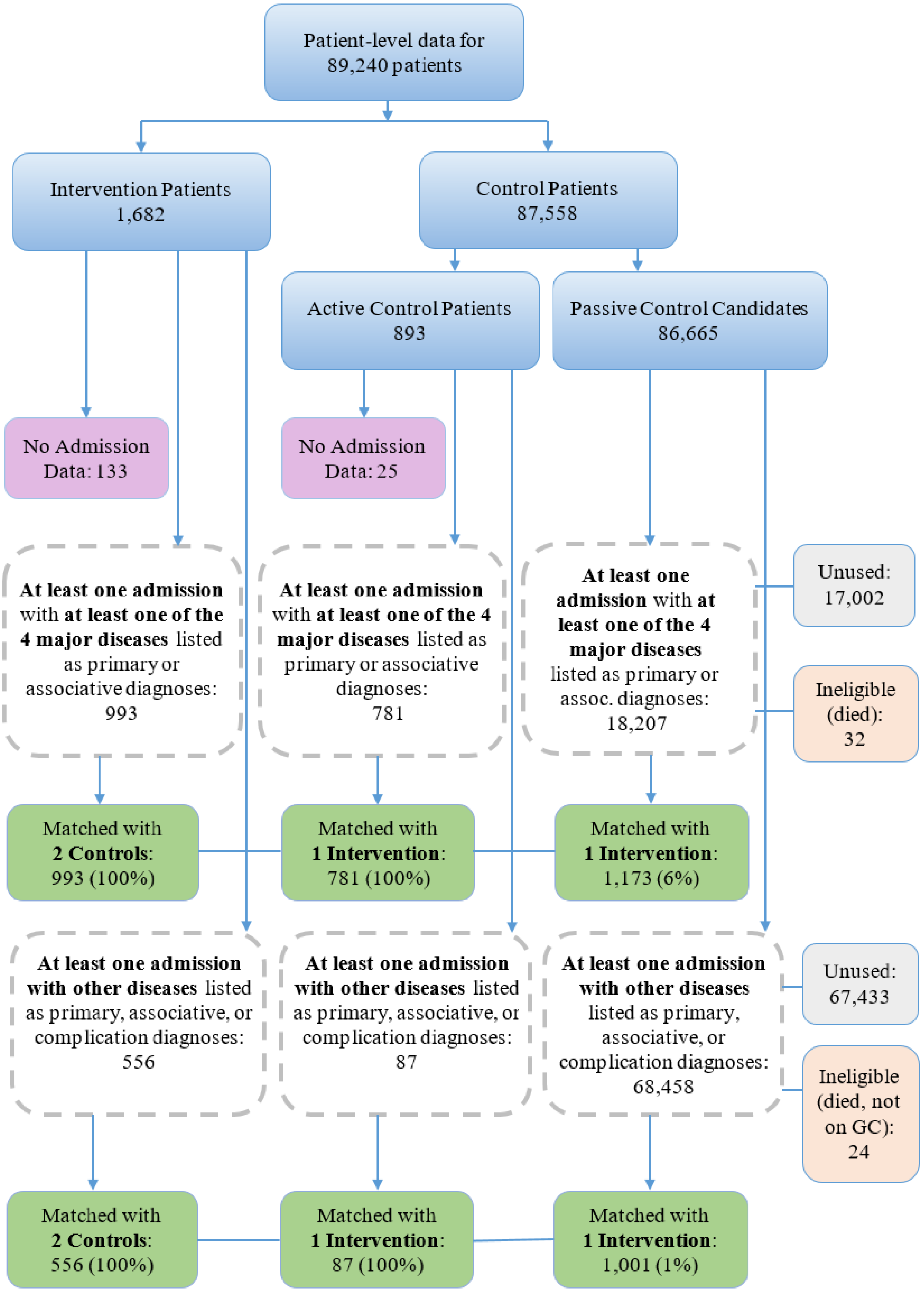

Figure S1. Control group matching flow chart

Step 2.

a. Direct radius matching was done, which was a multi-step process using the `radmatch` command in Stata. This user-written procedure shuffles the controls in a random order, then selects the required number of matches for each intervention participants based on a list of variables of interest and maximum tolerances (radius) for each. Radius of zero means exact match required.

Matching in sub-group (a) used the following list of variables based on presentations for those with the four main conditions:

1) gender (radius=0),
2) age in years in 2015 (radius=5),
3) heart disease (I20-52) as primary or associative diagnosis (radius=0),
4) chronic lower respiratory disease (J40–J47) (radius=0)
5) diabetes mellitus (E10–E14) as primary or associative diagnosis (radius=0),
6) renal failure (N17–N19) as primary or associative diagnosis (radius=0),
7) number of discharges (radius=0), and
8) percentage of discharges linked to potentially avoidable hospitalisations (radius=20).

The matching process was repeated (with slight adjustments) until 100% of intervention participants in sub-group (a) were matched with exactly two control participants.

b. Matching in the sub-group (b) used the following list of variables (including radius), based on common presentations in patients with other conditions:
    1) gender (radius=0),
    2) age in years in 2015 (radius=5),
    3) diseases of oesophagus, stomach and duodenum (K20-K31) as primary, associative or complication diagnosis (radius=0),
    4) hernia (K40-K46) as primary, associative or complication diagnosis (radius=0),
    5) other diseases of intestines (K55–K64) as primary, associative or complication diagnosis (radius=0),
    6) disorders of gallbladder, biliary tract and pancreas (K80–K87) as primary, associative or complication diagnosis (radius=0),
    7) other diseases of the digestive system (K90–K93) as primary, associative or complication diagnosis (radius=0),
    8) arthropathies (M00–M25) as primary, associative or complication diagnosis (radius=0),
    9) dorsopathies (M40–M54) as primary, associative or complication diagnosis (radius=0),
    10) soft tissue disorders (M60–M79) as primary, associative or complication diagnosis (radius=0),
    11) osteopathies and chondropathies (M80–M94) as primary, associative or complication diagnosis (radius=0),
    12) intentional self-harm (X60-X84) as primary, associative or complication diagnosis (radius=0),
    13) complications of medical and surgical care (Y40–Y84) as primary, associative or complication diagnosis (radius=0),
    14) supplementary factors related to causes of morbidity and mortality classified elsewhere (Y90-Y98) as primary, associative or complication diagnosis (radius=0),

15) falls (W00-W19) as primary, associative or complication diagnosis (radius=0),
16) number of discharges (radius=2), and
17) percentage of discharges linked to potentially avoidable hospitalisations (radius=20).

The matching process was repeated (with slight adjustments) until 100% of intervention participants in (b) were matched with exactly two control participants.

Step 3.
As recommended by Billot et al (2016), matching performance was investigated by comparing the baseline characteristics, healthcare utilisation and disease profile between the two study groups as shown in Table 1. Standardised difference was used as this is not influenced by sample size [43, 44]. Table 2 presents the participant characteristics by sub-group (a) and sub-group (b).

Table S1. Measures of equivalence: intervention and controls

	INTV n=1549	Control n=3042	Total N=4591	Std. diff.
Gender: females	822 (53%)	1608 (53%)	2430 (53%)	0.004
Age (years in 2015) [a]	70 (14)	69 (14)	69 (14)	0.040
No. of inpatient admissions (per patient) [b]	4 (2-8)	4 (2-7)	4 (2-7)	0.007
Potentially avoidablehosp. (%, per patient) [b]	0 (0-25)	0 (0-25)	0 (0-25)	0.010
Primary/associative diagnoses:				
diabetes mellitus (E10–14)	491 (32%)	972 (32%)	1463 (32%)	0.006
renal failure (N17–19)	320 (21%)	636 (21%)	956 (21%)	0.006
heart disease (I20–52)	528 (34%)	1004 (33%)	1532 (33%)	0.023
chronic lower respiratory disease (J40–47)	225 (15%)	450 (15%)	675 (15%)	0.008
Prim./assoc./compl. diagnoses (ICD-10):				
diseases of oesophagus, etc. (K20–K31)	236 (15%)	358 (12%)	594 (13%)	0.102
hernia (K40–K46)	88 (6%)	164 (5%)	252 (5%)	0.013
other diseases of intestines (K55–K64)	439 (28%)	782 (26%)	1221 (27%)	0.059
disorders of gallbladder, etc. (K80–K87)	86 (6%)	150 (5%)	236 (5%)	0.028
other digestive system diseases (K90–K93)	109 (7%)	214 (7%)	323 (7%)	<0.001
arthropathies (M00–M25)	266 (17%)	495 (16%)	761 (17%)	0.024
dorsopathies (M40–M54)	174 (11%)	308 (10%)	482 (10%)	0.036
soft tissue disorders (M60–M79)	126 (8%)	217 (7%)	343 (7%)	0.038
osteo- and chondropathies (M80–M94)	341 (22%)	608 (20%)	949 (21%)	0.050
complications of care (Y40–Y84)	536 (35%)	1033 (34%)	1569 (34%)	0.013
supplementary factors (Y90–Y98)	746 (48%)	1489 (49)	2235 (49%)	0.016
falls (W00–W19)	252 (16%)	568 (19%)	820 (18%)	0.064

[a] mean (standard deviation) shown; [b] median ($25^{th}/75^{th}$ percentiles) shown; data period: 01jul2012 to 30sep2016; hosp = hospitalisation; INTV = intervention; std. diff. = standardised difference; prim. = primary; assoc. = associative; compl. = complication;

Table S2. Matched participant characteristics

	Main diseases		Other diseases	
	Intervention n=993	Control n=1954	Intervention n=556	Control n=1088
Gender: females	487 (49)	956 (49)	335 (60)	652 (60)
Age group (years):				
up to 50	70 (7)	135 (7)	77 (14)	157 (14)
51 to 60	111 (11)	229 (12)	73 (13)	176 (16)
61 to 70	247 (25)	490 (25)	130 (23)	264 (24)
71 to 80	340 (34)	678 (35)	174 (31)	321 (29)
81 or higher	225 (23)	422 (22)	102 (18)	170 (16)
Utilisation outpatient appointments:				
nil	33 (3)	165 (8)	199 (18)	33 (6)
1 to 3	62 (6)	187 (10)	167 (15)	51 (9)
4 to 11	151 (15)	392 (20)	254 (23)	106 (19)
12 to 22	223 (22)	370 (19)	178 (16)	115 (21)
23 to 44	258 (26)	427 (22)	156 (14)	135 (24)
45 or more	266 (27)	413 (21)	134 (12)	116 (21)
Utilisation emergency presentations:				
nil	30 (3)	94 (5)	41 (7)	127 (12)
1	126 (13)	284 (15)	97 (17)	299 (27)
2	136 (14)	293 (15)	105 (19)	206 (19)
3 to 4	211 (21)	389 (20)	137 (25)	200 (18)
5 to 7	214 (22)	404 (21)	92 (17)	147 (14)
8 or more	276 (28)	490 (25)	84 (15)	109 (10)
length of stay (hours)[a c]	3.7 (3.1-4.6)	3.8 (3.0-4.7)	3.8 (2.9-4.8)	3.7 (2.8-4.6)
Utilisation inpatient admissions:				
nil	0 (0)	2 (0)	1 (0)	2 (0)
1	57 (6)	201 (10)	76 (14)	273 (25)
2 to 3	212 (21)	438 (22)	148 (27)	340 (31)
4 to 5	190 (19)	326 (17)	127 (23)	173 (16)
6 to 10	258 (26)	523 (27)	128 (23)	181 (17)
11 or more	276 (28)	464 (24)	76 (14)	119 (11)
length of stay (days)[a c]	2.2 (1.1-3.9)	2.2 (1.0-4.4)	1.3 (0.7-3.2)	1.1 (0.5-2.8)
- PPH[b c]	21%	20%	7%	6%
Chronic diseases (ICD-10 codes):				
diabetes mellitus (E10–E14)	499 (50)	985 (50)	22 (4)	16 (1)
heart (I20–I52)	570 (57)	1062 (54)	34 (6)	39 (4)
lower respiratory (J40–J47)	249 (25)	485 (25)	10 (2)	12 (1)
kidney (N17–19)	375 (38)	708 (36)	22 (4)	32 (3)
Number of above chronic diseases:				
nil	2 (0)	24 (1)	484 (87)	1008 (93)
1 of 4	506 (51)	1021 (52)	55 (10)	63 (6)
2 of 4	301 (30)	570 (29)	15 (3)	9 (1)
3 of 4	151 (15)	269 (14)	1 (0)	6 (1)
4 of 4	33 (3)	68 (3)	0 (0)	0 (0)

[a] median (25th and 75th percentiles) shown; prev. = preventable; [b] mean; sample period 01jul2012 to 30sep2017; frequencies and column percentages shown unless otherwise noted; [c] excluding patients with no events; PPH=potentially preventable hospitalisations

Reference

Billot, L., Corcoran, K., McDonald, A., Powell-Davies, G., & Feyer, A. M. Impact Evaluation of a System-Wide Chronic Disease Management Program on Health Service Utilisation: A Propensity-Matched Cohort Study. PLoS Med. 2016; 13(6), e1002035. doi:10.1371/journal.pmed.1002035
